# Developmental trajectories in infants and pre-school children with Neurofibromatosis 1

**DOI:** 10.1186/s13229-024-00621-5

**Published:** 2024-10-15

**Authors:** Hannah Slevin, Fiona Kehinde, Jannath Begum-Ali, Ceri Ellis, Emma Burkitt-Wright, Jonathan Green, Mark H. Johnson, Greg Pasco, Tony Charman, Emily J. H. Jones, Shruti Garg, the EDEN-STAARS team, Mary Agyapong, Tessel Bazelmans, Leila Dafner, Mutluhan Ersoy, Teodora Gliga, Amy Goodwin, Rianne Haartsen, Hanna Halkola, Alexandra Hendry, Rebecca Holman, Sarah Kalwarowsky, Sarah Lloyd-Fox, Luke Mason, Nisha Narvekar, Laura Pirazzoli, Chloë Taylor, Grace Vassallo, Judith Eelloo, D. Gareth Evans, Siobhan West, Eileen Hupton, Louise Robinson, Neeta Lakhani, Brian Wilson, Deborah Osio, Charles Shaw-Smith, Natalie Canham, Saba Sharif

**Affiliations:** 1 Division of Psychology and Mental Health, Faculty of Biology, Medicine and Health, University of Manchester, Manchester, UK; 2 Centre for Brain and Cognitive Development and Department of Psychological Sciences, Birkbeck, University of London, London, UK; 3 Division of Psychology, Communication and Human Neuroscience, Faculty of Biology, Medicine and Health, University of Manchester, Manchester, UK; 4 Manchester Centre for Genomic Medicine, Manchester University NHS Foundation Trust, Manchester, UK; 5 Faculty of Biology, Medicine and Health, University of Manchester, Manchester, UK; 6 Harrington Building, Royal Manchester Children’s Hospital, Oxford Rd, Manchester, M13 9WL UK; 7 Department of Psychology, University of Cambridge, Cambridge, UK; 8 Psychology Department, Institute of Psychiatry, King’s College London, Psychology & Neuroscience, London, UK

**Keywords:** Neurofibromatosis, NF1, Trajectories, Cohort, Autism, ADHD, Children

## Abstract

**Background:**

Children with Neurofibromatosis 1 (NF1) show cognitive, behavioural and social differences compared to their peers. However, the age and sequence at which these differences begin to emerge is not fully understood. This prospective cohort study examines the cognitive, behavioural, ADHD trait and autism symptom development in infant and pre-school children with NF1 compared with typically developing (TD) children without a family history of neurodevelopmental conditions.

**Methods:**

Data from standardised tests was gathered at 5, 10, 14, 24 and 36 months of age (NF1 n = 35, TD n = 29). Developmental trajectories of cognitive (Mullen Scales of Early Learning, MSEL) and adaptive behavioural (Vineland Adaptive Behavior Scales, VABS) development from 5 to 36 months were analysed using linear mixed modelling. Measures of ADHD (Child Behavior Checklist) and autism traits (ADOS-2, BOSA-MV and ADI-R) were assessed at 24 and 36 months.

**Results:**

The developmental trajectory of cognitive skills (all domains of the MSEL) and behavioural skills (four domains of the VABS) differed significantly between NF1 and TD groups. Post-hoc tests demonstrated that the NF1 participants scored significantly lower than TD participants at 24 months on all MSEL and VABS domains. The NF1 cohort demonstrated higher mean autism and ADHD traits at 24 months and 14% of the NF1 cohort met a research diagnostic classification for autism at 36 months.

**Limitations:**

The study has a relatively small sample size due to variable retention and rolling recruitment. Due to limitations imposed by the COVID-19 pandemic, we utilised the Brief Observation of Symptoms of Autism for Minimally Verbal children (BOSA-MV) for some participants, which was administered online and may not gather as accurate a picture of traits as ADOS-2. The BOSA-MV was utilised for 41% of participants with NF1 at 36 months compared to 11% at 24 months. This may explain the reduction in the percentage of children with NF1 that met autism criteria at 36 months.

**Conclusions:**

By 24 months of age, the NF1 cohort show lower cognitive skills and adaptive behaviour and higher levels of autism and ADHD traits as compared to TD children. This has implications for developmental monitoring and referral for early interventions.

**Trial registration:**

Not applicable.

**Supplementary Information:**

The online version contains supplementary material available at 10.1186/s13229-024-00621-5.

## Introduction

Neurofibromatosis 1 (NF1) is a single gene neurocutaneous condition, with a birth incidence of 1 in 2700 [1]. 50% of cases arise through autosomal dominant inheritance from a parent, and 50% via a sporadic pathogenic variant of the tumour-suppressor NF1 gene on chromosome 17q11.2. This gene codes for the protein neurofibromin, which plays a role in regulating neuronal cell development [2]. NF1 is characterised by phenotypic variability, and clinical diagnosis is based on the revised National Institute of Health (NIH) criteria [3].

Many children with NF1 experience cognitive and adaptive behavioural difficulties. Overall intellectual ability is only slightly lower than children who do not have NF1, however specific cognitive deficits impact perceptual (particularly visuospatial) skills, executive functioning, adaptive behaviour, attention and motor skills [4, 5]. Research also indicates high rates of co-occurring neurodevelopmental conditions such as Autism Spectrum Disorder (henceforth referred to as autism) (29%) and Attention Deficit Hyperactivity Disorder (ADHD) (50%) [6, 7] .

The majority of studies analysing the cognitive, social and behavioural phenotype in NF1 have concentrated on school-aged children [8]. Several cross-sectional studies have focused on the pre-school period, which consistently demonstrate that young children with NF1 show cognitive, behavioural and social differences compared to age-matched controls [2, 4, 9–14]. The majority of these studies examined children from the age of 3 years, although one included children as young as 7 months of age [14].

However, the age and sequence at which these differences begin to emerge is not fully understood. Understanding this is crucial for identifying early markers of later neurodevelopmental outcomes. Neurodevelopmental conditions such as autism and ADHD likely arise from complex interactions between the genome, brain and environment, and diagnoses tend to be made in school-aged children [15]. However, there is evidence to suggest that early parent-administered social communication interventions around 7–10 months of age could reduce the later behavioural manifestations of autism [16]. There are also clinical implications for screening and intervention in children with NF1, particularly educational planning [15] .

Two previous studies have analysed trajectories of cognitive development in toddlers [17, 18]. Lorenzo et al. assessed the early development of 39 children with NF1 aged 21, 30 and 40 months compared with matched controls; the NF1 cohort demonstrated lower cognitive functioning than controls [17]. Wessel et al. assessed 124 children with NF1 aged 0–8 years of age using parental report measures. Gross and fine motor delays were found to emerge aged 3–5 years, whereas academic delays tended to present at a later age [18]. However, the majority of the cohort had just one assessment.

A previous paper from our group examined trajectories of cognitive and adaptive behavioural development in infants with NF1 aged 5–14 months compared with typically developing (TD) children [15]. At this early stage in development, no group differences were observed in trajectories of cognitive and adaptive functioning, nor differences on social communication measures. However, early differences in neural processing including auditory processing and excitation/inhibition balance were observed, both of which were related to later autistic traits [19, 20] .

It was important to examine children with NF1 from 5 months of age, because of the lack of evidence about how developmental trajectories evolve in infancy in children with NF1. An earlier case series examined a number of the current sample of infants at 10 months of age [21]. This suggested early differences in motor and communication skills, highlighting the importance of studying development in the first year of life in children with NF1.

Our study aim is to examine the early cognitive, behavioural, social and ADHD trait development of infants and pre-school aged children with NF1, compared with a cohort of TD children. Our objectives were to build on the earlier work of our team [15] by examining longitudinal trajectories of cognitive and behavioural development from 5–36 months, and ADHD trait development at 24 and 36 months. We also aimed to examine emergence of autism traits at 24 and 36 months. This study captures the natural progression of the children, and they did not take part in any interventions within the remits of this study.

We hypothesised that children with NF1 would have lower cognitive and adaptive functioning over time, and higher levels of ADHD and autism traits compared with TD children. Based on our previous work [15], we interrogate whether differences begin to emerge at the age of 24 or 36 months. To our knowledge, this is the first study to examine cognitive and behavioural development from infancy in children with NF1 using both parental report and objective assessment measures. This prospective approach is critical in avoiding ascertainment bias seen with older children, where participants may be more likely to participate if they are experiencing developmental delays.

## Methods

The Early Development in NF1 (EDEN) study is a UK-based prospective longitudinal cohort study investigating early development in infants and children with NF1. The behavioural measures used in our study were part of a more comprehensive experimental protocol used for the EDEN study, and our data formed a proportion of the results obtained from EDEN. Our previous paper also describes the methods used in the EDEN study [15] .

### Recruitment

Participants were enrolled through regional genetic centres and NF1 charities. Rolling recruitment was conducted between 2016 and 2019. Participants in the TD group were enrolled from a volunteer database for the Studying Autism and ADHD in at Risk Siblings (STAARS) study at the Centre for Brain and Cognitive Development, Birkbeck, University of London. These children had typical development and had not been diagnosed with a developmental disorder. The sample size calculation derived from our previous work comparing infants at high likelihood of developing autism to controls (e.g. n = 17, η^2^ = 0.17; *n* = 19, η^2^ = 0.16) [22, 23]. However it is important to note that this was based on detecting EEG biomarker differences rather than the behavioural measures utilised in this study.

Inclusion criteria for the NF1 cohort included (a) infant under 14 months of age at the time of recruitment (b) NF1 diagnosed via testing of cord blood samples or clinical diagnosis.

Inclusion criteria for the TD group included (a) infant under 14 months of age at the time of recruitment (b) no first-degree relatives with known genetic conditions, autism or ADHD (c) no parent-reported developmental issues in the child (d) full-term birth (gestational age at least 36 weeks).

Exclusion criteria for both groups included (a) conditions which might make it difficult for the infant to participate, such as physical complications of NF1 (b) significant hearing or visual impairments (c) significant prematurity (d) parents with significant learning difficulties or who were unable to give informed consent.

To offer maximum flexibility for participants, recruitment was offered up to the age of 14 months. Retention was variable across visits, which meant that the sample size varied at different assessment time points. However, participants could rejoin for later assessments if they were unable to attend at a particular timepoint.

35 children with NF1 and 29 TD participants were enrolled. 8 NF1 participants and 15 TD participants completed all 5 visits, 12 NF1 participants and 9 TD participants completed 4 visits, 9 NF1 and 2 TD participants completed 3 visits, 3 NF1 and 0 TD participants completed 2 visits, and 3 NF1 and 3 TD participants completed 1 visit. Further information on study numbers and attrition is outlined in the supplementary material (Supplementary Fig. 1).

### Testing

Participants were assessed at 5, 10, 14, 24 and 36 months of age. The study assessments took place at the Division of Psychology and Mental Health, University of Manchester, and the Centre for Brain and Cognitive Development, Birkbeck, University of London. The NF1 participants at 5, 10, 14 and 24 months were tested at Birkbeck, and the University of Manchester at 36 months.

Prior written informed consent was obtained from the parent. Testing took place if the child was physically well and content. Assessments were carried out over 2 days for infants at 5, 10 and 14 months, to account for breaks and sleep schedules, and over one full day for the older participants at 24 and 36 months. Participant families were provided with reimbursement for expenses for travel, food and overnight stay in a hotel if required. A £20 gift card was offered as an incentive for each visit completion.

### Measures

Table 1 summarises the measures used at each time point.

**Table 1:** Measures administered at each time point

5 Months	10 Months	14 Months	24 months	36 months
Mullen Scales of Early Learning (MSEL)	Mullen Scales of Early Learning (MSEL)	Mullen Scales of Early Learning (MSEL)	Mullen Scales of Early Learning (MSEL)	Mullen Scales of Early Learning (MSEL)
Vineland Adaptive Behavior Scales (VABS)	Vineland Adaptive Behavior Scales (VABS)	Vineland Adaptive Behavior Scales (VABS)	Vineland Adaptive Behavior Scales (VABS)	Vineland Adaptive Behavior Scales (VABS)
			Child Behavior Checklist (CBCL)	Child Behavior Checklist (CBCL)
				Autism Diagnostic Interview-Revised (ADI-R)
			Autism Diagnostic Observation Schedule-2 (ADOS-2)	Autism Diagnostic Observation Schedule-2 (ADOS-2)
			OR	OR
			Brief Observation of Symptoms of Autism (BOSA-MV)	Brief Observation of Symptoms of Autism (BOSA-MV)

#### Maternal education

Maternal education was collected as part of a larger questionnaire ascertaining demographic factors. It was classified as either primary, secondary, undergraduate or postgraduate (1,2,3 or 4) (Table 2). We focused on maternal, rather than paternal, education level due to evidence suggesting that among core domains of socio-economic status (employment, income and education), maternal education is most strongly associated with a child’s cognitive development [24]. Maternal education has been shown to be significantly associated with trajectories of cognitive and adaptive functioning at 5, 10 and 14 months in this population [15] .

**Table 2:** Demographic characteristics at each Timepoint

	5 Months				10 Months				14 Months				24 months				36 months			
	TD group		NF1 Group		TD group		NF1 Group		TD group		NF1 Group		TD group		NF1 Group		TD group		NF1 Group	
	*n*		*n*		*n*		*n*		*n*		*n*		*n*		*n*		*n*		*n*	
Age at time of testing in days: Mean [SD]	26	179.19 [14.05]	15	194.73 [17.85]	27	321.93 [16.70]	23	327 [17.11]	23	447.7 [18.37]	27	449.74 [23.41]	24	762.25 [36.07]	29	795.41 [90.98]	20	1135.45 [55.31]	30	1255.93 [164.87]
Sex: Males	26	17	15	8	27	16	23	12	23	13	27	13	24	13	29	14	20	12	30	14
Sex: Females		9		7		11		11		10		14		11		15		8		16
Maternal education: Secondary	23	2	12	8	26	2	23	15	23	2	25	14	23	2	26	15	19	1	26	16
Maternal education: Undergraduate		8		2		10		5		7		8		8		8		7		7
Maternal education: Postgraduate		13		2		14		3		14		3		13		3		11		3

#### Cognitive and adaptive behavioural skills

The Mullen Scales of Early Learning (MSEL) [25], a standardised assessment for children aged up to 68 months, was used to assess cognitive functioning at all five time points. Five domains were assessed, including Visual Reception, Fine Motor, Receptive Language, Expressive Language skills (all measured as T-scores) and an Early Learning Composite (ELC) (Standard Score). T-scores range from 20 to 80 and the ELC standard score range from 49 to 155.

The Vineland Adaptive Behavior Scales (VABS) Third Edition [26], a parent-report questionnaire, was used to assess adaptive behavioural skills at all five time points. The standard scores of five domains were assessed, including Communication, Daily Living Skills, Socialisation, Motor skills and an Adaptive Behavior Composite score. Standard scores range from 20 to 160.

#### ADHD traits

The Child Behavior Checklist (CBCL) for ages 1.5–5 [27], a parent-report questionnaire, was used to assess ADHD traits (inattention/hyperactivity) at 24 and 36 month time points. T-scores were used for the DSM-orientated Attention Deficit Hyperactivity problems scale. T-scores of 70 are in the clinically significant range, and 65–69 are considered borderline [28] .

#### Autism traits

The Autism Diagnostic Observation Schedule (ADOS-2), a semi-structured assessment of social communication, social interaction and imaginative play for individuals suspected to have autism [29, 30], was utilised at 24 and 36 months. Based on the expressive language ability of the participants, either the Toddler module, Module 1 or Module 2 of the ADOS-2 were used at 24 and 36 months (Table 3).

**Table 3:** Descriptive statistics for ADI-R, ADOS, BOSA and CBCL

	24 months				36 months			
	TD group		NF1 Group		TD group		NF1 Group	
	*n*	*Mean [SD]*	*n*	*Mean [SD]*	*n*	*Mean [SD]*	*n*	*Mean[SD]*
*Autism Diagnostic Interview—Revised (ADI-R) Diagnostic Algorithm*								
Qualitative Abnormalities in Reciprocal Social Interaction (A)		N/A		N/A	18	0.94 [1.00]	30	5.23 [4.79]
Qualitative Abnormalities in Communication (B)					18	0.67 [0.97]	30	5.03 [4.42]
Restricted, Repetitive and Stereotyped Patterns of Behaviour (C)					18	0.39 [0.61]	30	2.13 [2.60]
Abnormality of Development Evident at or Before 36 Months (D)					18	0.06 [0.24]	30	1.83 [1.34]
Meeting threshold for autism on ADI-R					18	0 [0%)]	30	5 [17%]
Not meeting threshold for autism on ADI-R					18	18 [100%]	30	25 [83%]
*Autism Diagnostic Observation Schedule-2 (ADOS-2)*								
Module (n) (Toddler/Module 1/Module 2)	24	24/0/0	24	21/3/0	20	0/0/20	17	0/16/1
Total scores	24	3.67 [1.95]	24	8.08 [6.64]	20	4.35 [3.42]	17	4.47 [3.86]
Social Affect scores	24	3.13 [1.80]	24	6.83 [5.75]	20	3.60 [2.84]	17	3.65 [3.02]
Restrictive and Repetitive Behaviour scores	24	0.54 [0.72]	24	1.25 [1.29]	20	0.80 [0.95]	17	0.82 [1.24]
Number reaching threshold for Autism Spectrum Disorder or Autism on ADOS-2	24	0 [0%]	24	11 [46%]	20	5 [25%]	17	4 [24%]
*Brief Observation of Symptoms of Autism (BOSA-MV)*								
Module (Toddler/Module 1)	0	0/0	3	2/1	0	0/0	12	0/12
Total scores			3	12.33 [4.93]			12	8.17 [2.66]
Algorithm scores			3	6.00 [2.00]			12	4.42 [1.44]
Number reaching threshold for Autism Spectrum Disorder on the BOSA			3	2 [67%]			12	5 [42%]
*ADOS/BOSA combined*								
Number of participants reaching threshold for Autism	24	0 [0%]	27	13 [48%]	20	5 [25%]	29	9 [31%]
Number of participants not reaching threshold for Autism	24	24 [100%]	27	14 [52%]	20	15 [75%]	29	20 [69%]
Number of participants reaching threshold for Autism on both ADI-R and ADOS/BOSA		N/A		N/A	18	0 [0%]	29	4 [14%]
*Child Behavior Checklist ADHD subscale*								
T score	22	51.86 [2.49]	27	55.56 [7.84]	21	51.10 [1.45]	28	57.14 [6.88]

All three ADOS-2 modules provide a score for the domains of Social Affect and Restricted and Repetitive Behavior, followed by a total score. For the Toddler module, separate algorithms are based on age and language ability. For our study, children at 24 months who produced fewer than 5 words during the ADOS-2 received the non-verbal 21–30 months algorithm, and children who produced 5 or more words received the verbal 21–30 months algorithm. Total scores were classified into ‘levels of concern’ for autism: no concern, mild-to-moderate concern (a score of 10 + for the non-verbal algorithm and 8 + for the verbal algorithm) or moderate-to-severe concern (a score of 14 + for the non-verbal algorithm and 12 + for the verbal algorithm). Luyster et al. suggest that at least 95% of children with Autism Spectrum Disorder and no more than 10% of typically developing children would fall into the two groups suggesting clinical concern on the ADOS Toddler module (mild-to-moderate and moderate-to-severe). This gives an instrumental sensitivity of at least 95% and a specificity of more than 90% [30] .

For Module 1, children with some language who gain a score of 8 + receive a classification of autism spectrum, and a score of 12 + gives a classification of autism. For children with few to no words, a score of 11 + gives a classification of autism spectrum and a score of 16 + gives a classification of autism. For Module 2, children less than 5 years of age who receive a total score of 7 + receive a classification of autism spectrum and a score of 10 + gives a classification of autism. Comparison scores can also be calculated to indicate level of autism-related symptoms, although analysis of this data was beyond the scope of this paper.

Coding was carried out from videos, with an inter-rater reliability of 79.1% for the NF1 cohort.

The ADOS-2 was administered from 24 months of age. Although the ADOS-2 Toddler module can be used for children from 12 months of age, Luyster et al. (2009) recognised in their development of the instrument that their final sample would include very few children in the autism group at this lower cutoff due to the frequency of developmental delay in children with autism [30] .

During 2020–2022, the Covid-19 pandemic required some assessments to be carried out virtually, as a result of social distancing legislation. For some participants, the Brief Observation of Symptoms of Autism for Minimally Verbal children (BOSA-MV) was utilised. This is an observational measure designed to be administered remotely [31]. Based on the expressive language ability of the participants, either the Toddler module or Module 1 of the BOSA-MV were used at 24 and 36 months (Table 3).

Both BOSA-MV modules provide a score for the domains of Impairment in Social Communication and Social Interaction, and Restricted and Repetitive Behaviors, followed by a total score. This gives a range of concern for autism of little to no concern, mild-to-moderate concern and moderate-to-severe concern. Dow et al. recommend a cut-off of 6 for Autism Spectrum Disorder for the BOSA-MV toddler module (corresponding with the moderate-to-severe concern category) and a score of 5 as a cut-off for the BOSA-MV Module 1 (corresponding with the mild-to-moderate or moderate-to-severe category) [31]. For the Toddler module, this gives an instrumental sensitivity of 96% and a specificity of 83%. For Module 1, this provides a sensitivity of 91% and a specificity of 100% (although the authors acknowledge that their non-autism sample was small when developing the BOSA-MV) [31].

Table 3 outlines the participant numbers at each timepoint who were administered ADOS-2 versus BOSA-MV.

The Autism Diagnostic Interview-Revised (ADI-R), an investigator-based semi-structured interview for parents [32], was carried out at 36 months. Scoring is based on two algorithms, depending on whether the subject is verbal or non-verbal. Four subscales are produced: A—qualitative abnormalities in reciprocal social interaction, B—qualitative abnormalities in communication, C—restricted, repetitive and stereotyped patterns of behaviour and D—abnormalities of development evident at or before 36 months. Each subscale has a cut-off for autism (A = 10, B = 8 if verbal and 7 if non-verbal, C = 3 and D = 1) [32] .

The ADI-R was utilised at the 36 month time point. Psychometric analyses have determined that for children over 36 months of age, the algorithms differentiate children with autism from those with non-spectrum disorders with a high sensitivity and specificity of over 90% [33] .

The ADOS-2/BOSA and ADI-R assessors were not blind to the participant’s group (NF1 versus TD children), however videos and interviews were double coded and this second coder was blind as to the participant’s condition.

### Classification of autism

In our paper, the following thresholds are used on the ADOS-2 and BOSA-MV to determine autism traits at 24 months of age:

- ‘mild-to-moderate’ or ‘moderate-to-severe’ concern on the ADOS-2 Toddler module.

OR

- ‘autism-spectrum’ or ‘autism’ on ADOS-2 Module 1.

OR

- a score of 6 on the BOSA-MV toddler module

OR

- or a score of 5 on the BOSA-MV Module 1.

Participants were assigned a research instrumental classification of autism at 36 months of age if they met threshold on either the ADOS-2 or BOSA-MV, in addition to meeting threshold on the ADI-R:

- ‘autism-spectrum’ or ‘autism’ on ADOS-2 Module 1.

OR

- ‘autism-spectrum’ or ‘autism’ on ADOS-2 Module 2.

OR

- a cut off of 5 on the BOSA-MV Module 1.

ALONG WITH

- meeting threshold for subscale A and coming within one point of B, or meeting threshold for B and coming within one point of A on the ADI-R, as suggested by Risi et al. [34].

Risi et al. provide a rationale for combining the ADI-R with the ADOS-2 at 36 months of age, giving a sensitivity of 61.1% for autism detection and a specificity of 87.7% for the combination of the ADOS-2 with ADI-R criteria for A and B as outlined above [34]. To our knowledge, there have been no sensitivity and specificity estimates for the combination of BOSA-MV and ADI-R, due to the relative recency of the BOSA-MV.

A research classification of autism was not given at 24 months, as the ADI-R was not utilised at 24 months in this study, and best diagnostic practice combines parent report with objective assessments.

### Statistical analyses

Statistical analyses were performed using IBM SPSS Statistics 28.0.0.0. Linear mixed modelling was used to analyse the change in cognition (MSEL), adaptive behaviour (VABS) and ADHD traits (CBCL DSM-ADHD subscale) over time. For each subscale, overall group differences were modelled using fixed effects (group, timepoint and sex) and random effects (ID – individual variation). Maternal education was included as a co-variate within the model [15] .

In all models, sex was non-significant (Table 4**)**. Age in days was not included in the model as a fixed effect, as this had already been corrected for by using age-corrected T scores (MSEL and CBCL) and age-corrected standard scores (VABS). Post hoc T-tests were carried out to further explore group differences on the MSEL and VABS at each timepoint.

**Table 4:** Linear mixed modelling F statistic and p values of fixed effects for MSEL, VABS AND CBCL

	MSEL: Visual Reception			Fine Motor			Receptive Language			Expressive Language			Early Learning Composite		
	*df*	*F*	*p*	*df*	*F*	*p*	*df*	*F*	*p*	*df*	*F*	*p*	*df*	*F*	*p*
Intercept	1, 56	3567.94	< .001*	1,53	1786.79	< .001*	1, 53	3036.60	< .001*	1, 56	2590.40	< .001*	1, 53	4364.92	< .001*
Group	1, 56	84.72	< .001*	1,53	64.16	< .001*	1, 53	111.37	< .001*	1, 57	36.59	< .001*	1, 53	85.30	< .001*
Timepoint	4, 178	38.46	< .001*	4, 174	7.11	< .001*	4, 177	53.75	< .001*	4, 179	19.57	< .001*	4, 170	61.97	< .001*
Sex	1, 54	0.18	.676	1, 51	0.17	.679	1, 50	0.38	.541	1, 54	1.33	.254	1, 51	0.20	.657
Group x Timepoint	4, 178	30.83	< .001*	4, 174	13.65	< .001*	4, 177	23.51	< .001*	4, 179	20.03	< .001*	4, 170	41.30	< .001*

	VABS: Communication			Daily Living Skills			Socialization			Motor Skills			Vineland Adaptive Behaviour Composite		
	*df*	*F*	*p*	*df*	*F*	*p*	*df*	*F*	*p*	*df*	*F*	*p*	*df*	*F*	*p*
Intercept	1, 50	7107.57	< .001*	1, 55	8034.63	< .001*	1, 59	7396.78	< .001*	1, 54	5814.12	< .001*	1, 57	6986.46	< .001*
Group	1, 50	14.15	< .001*	1, 55	5.60	.022*	1, 59	2.02	.160	1, 54	22.55	< .001*	1, 57	13.92	< .001*
Timepoint	4, 148	10.97	< .001*	4, 156	9.57	< .001*	4, 150	3.72	.006*	4, 149	12.24	< .001*	4, 143	8.24	< .001*
Sex	1, 48	1.39	.245	1, 53	1.84	.180	1, 58	2.34	.132	1, 53	.52	.475	1, 56	2.29	.136
Group x Timepoint	4, 148	6.78	< .001*	4, 156	3.95	.004*	4, 150	5.31	< .001*	4, 149	2.65	.035*	4, 143	8.22	< .001*

	CBCL DSM-ADHD Subscale														
	*df*	*F*	*p*												
Intercept	1,50	6252.67	< .001*												
Group	1, 50	11.21	.002*												
Timepoint	1, 46	0.02	.885												
Sex	1, 50	1.97	.167												
Group x Timepoint	1, 46	0.85	.361												

Maternal education was included in all models as a covariate. df = degrees of freedom, F statistic, *p* value, * = significant results

Missing data was imputed using the maximum likelihood option. Earlier time points were imputed for children that joined later in the study and data from subsequent missed sessions were also imputed. 29% of the NF1 dataset was imputed and 17% of the TD dataset was imputed (Supplementary Fig. 1).

A p value of below 0.05 was determined to be significant for the MSEL and VABS. For post-hoc tests, a Bonferroni corrected p value of below 0.01 was determined to be significant. This was based on the use of T-tests at 5 time points for each measure (0.05/5).

Pearson Chi-squared tests were carried out for proportion of participants meeting autism threshold on the ADOS-2/BOSA/ADI-R. Mann–Whitney non-parametric analyses were used to compare ADI-R subscale means due to non-normality.

## Results

35 children with NF1 and 29 TD participants were enrolled. Table 2 provides further details of the demographic characteristics and number of participants per measure. 32 of the participants with NF1 had an inherited pathogenic variant, 2 arose de novo and 1 had an unknown mechanism of inheritance.

Maternal education significantly differed between groups, with mothers of TD children more likely to have a post-graduate education (Median NF1: 2, TD: 4 X^2^ (2) = 19.79, *p* < 0.001).

The NF1 group was significantly older than the TD group at 5 months (t = 3.09, 95% CI 5.36 to 25.72 days, *p* = 0.004, *d* 1.00) and at 36 months (t = 3.70, 95% CI 54.60 to 186.36 days, *p* =  < 0.001, *d* 0.91).

### Trajectories of cognitive and adaptive behavioural development

Table 4 summarises the results of the linear mixed models for each of the measures described below. The mean and standard deviation for T-scores and standard scores are presented in Supplementary Table 1, and post-hoc statistics are detailed in Supplementary Table 2. The estimated marginal means, adjusting for maternal education as a co-variate, are displayed in Supplementary Table 3.

**Fig. 1:**
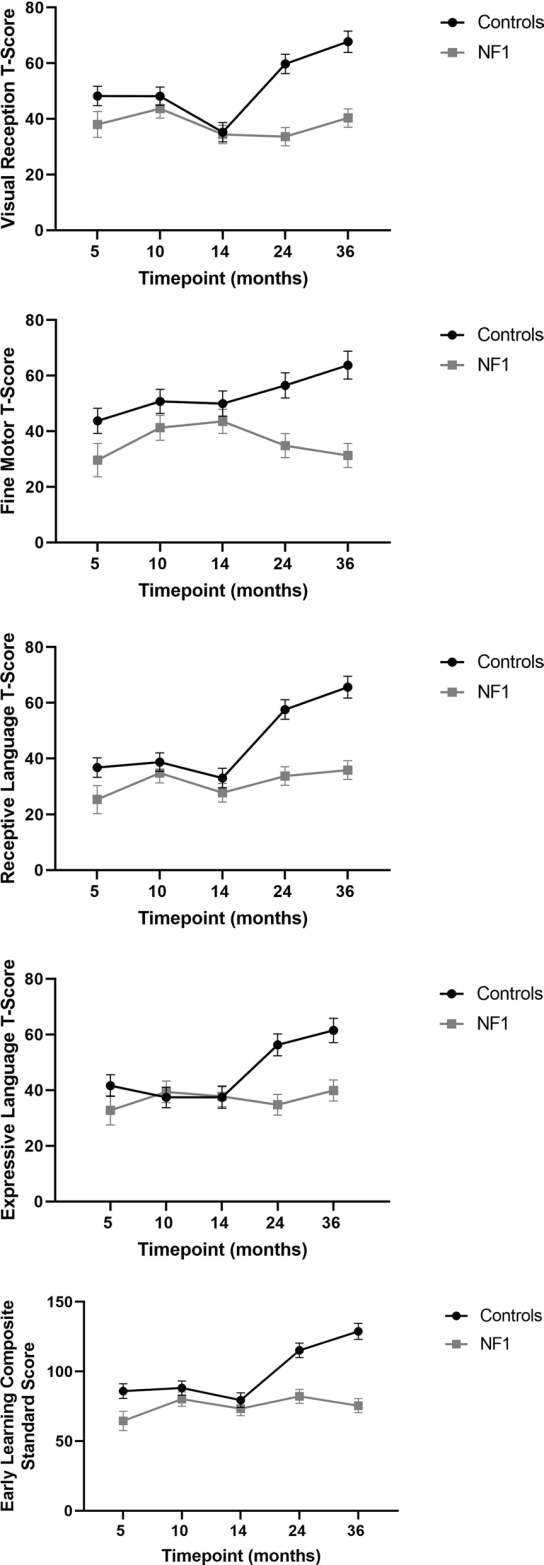
Estimated marginal mean scores on the MSEL (Error bars represent 95% confidence intervals)

On the MSEL, the developmental trajectory of children with NF1 differed significantly compared to the trajectory of TD children across all subscales (Visual Reception, Fine Motor, Receptive Language, Expressive Language, Early Learning Composite), with slower progress in the NF1 group (Fig. 1). Pairwise comparisons showed significant differences between the NF and TD groups on all MSEL domains at 5, 24 and 36 months (Supplementary Table 2). Only lower Fine Motor skills were observed at 10 months in the NF1 group, with no differences between the groups at 14 months.

**Fig. 2:**
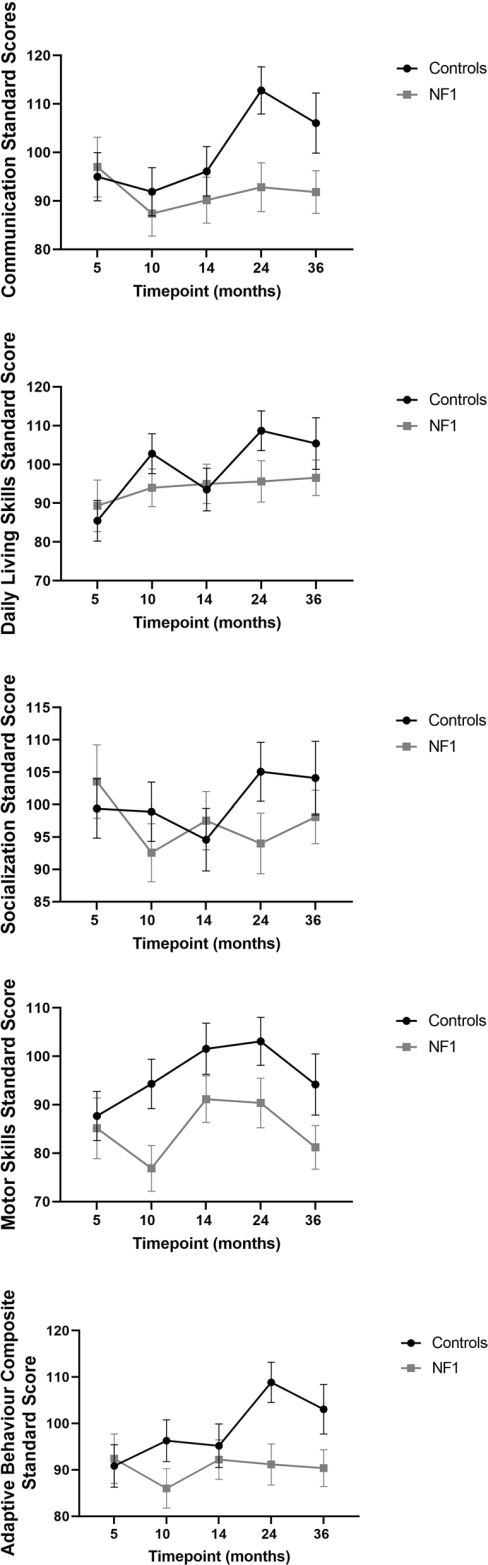
Estimated marginal mean scores on the VABS (Error bars represent 95% confidence intervals)

The trajectories of adaptive behavioural skills development were significantly different in NF1 when compared with TD children on four subscales of the VABS; Communication abilities, Daily living skills, Motor Skills and the Adaptive Behavior Composite domain, with slower progress in the NF1 group. However, there was no significant difference between children with NF1 and TD children on the Socialization domain, apart from at the 24 month time-point (Fig. 2). Pairwise comparisons showed that the NF1 participants scored significantly lower than TD participants in Daily living skills, Motor skills and the Adaptive Behavior Composite at 10 months, on all VABS domains at 24 months, but only on communication at 36 months (Supplementary Table 2).

### ADHD trait development

**Fig. 3:**
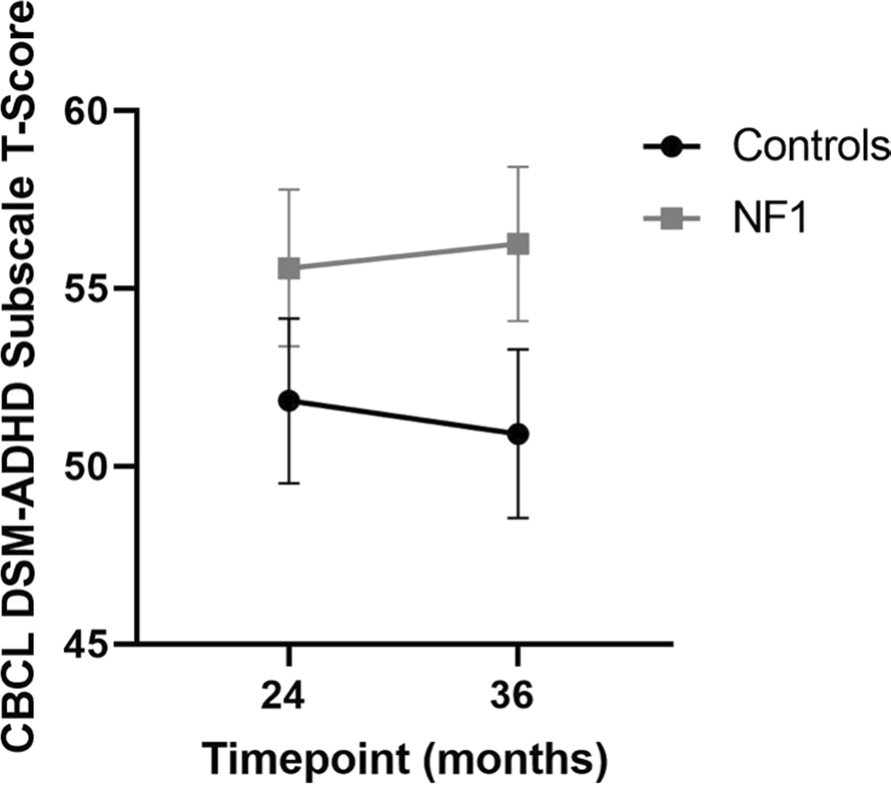
Estimated marginal means of T-scores on CBCL-DSM ADHD Subscale (Error bars represent 95% confidence intervals)

Children with NF1 showed higher mean levels of ADHD traits on the CBCL DSM-ADHD subscale than TD children **(**Fig. 3**, **Table 4**)**. 10.5% of NF1 participants at 36 months had a T-score over 65, suggesting borderline or clinically significant ADHD traits. There was no significant difference in CBCL score between male and female participants.

### Autism trait development

Mean total ADOS-2 scores were higher in the NF1 group as compared to the TD group. 48% of NF1 participants at 24 months scored above threshold for autism on the ADOS-2 or BOSA-MV instruments, compared to 0% of TD children (X^2^ (1) = 15.51, *p* < 0.001) (Table 3). More male than female participants reached instrumental threshold for autism in the NF1 group (X^2^ (1) = 6.24, *p* = 0.01).

At 36 months, 31% of participants in the NF1 group scored above threshold for autism on the ADOS-2 or BOSA. This was not statistically significant (X^2^ (1) = 0.21, *p* = 0.65), as 25% of TD participants also scored above threshold (although no TD participants gained an eventual research classification of autism when the ADI-R was considered) (Table 3). There were no statistically significant sex differences between the autism and non-autism participants in either the NF1 (X^2^ (1) = 2.52, *p* = 0.11) or TD groups (X^2^ (1) = 1.11, *p* = 0.29).

**Fig. 4:**
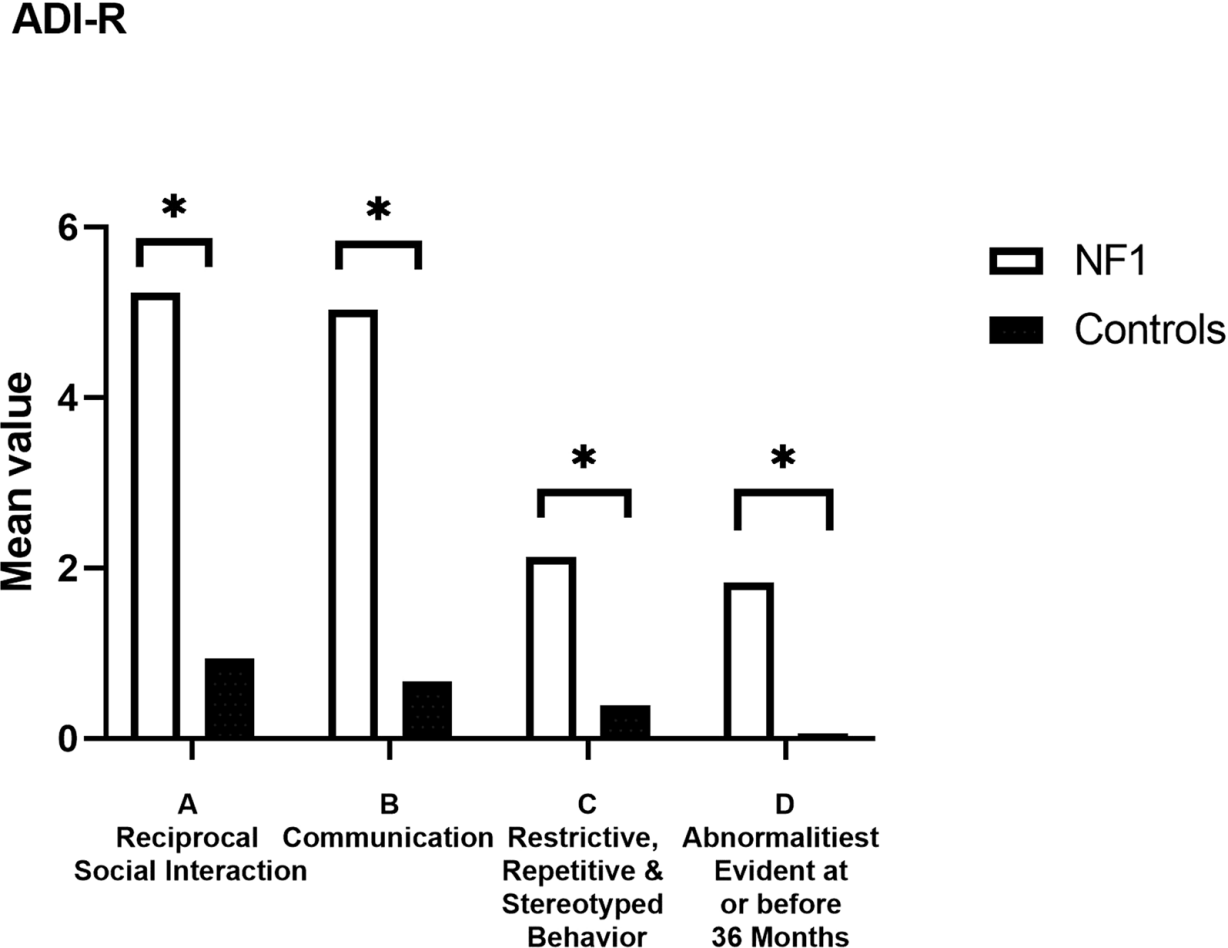
Mean values for subscales A-D on the Autism Diagnostic Interview-Revised (ADI-R). Significant differences denoted by *

On the ADI-R at 36 months, there were significant differences, with higher mean scores on each of the 4 subscales in NF1 compared to TD participants (*p* < 0.001) (Fig. 4, Table 3). Qualitative abnormalities in reciprocal social interaction differed significantly between NF1 and TD participants (U = 83.50, z =− 4.03, *p* =  < 0.001). Qualitative abnormalities in communication also significantly differed (U = 88.0, z =− 3.95, *p* =  < 0.001). Restricted, repetitive and stereotyped patterns of behaviour differed significantly between NF1 and TD participants (U = 126.00, z =− 3.23, *p* =  < 0.001). Finally, abnormalities of development evident at or before 36 months of age differed significantly (U = 59.50, z =− 4.79, *p* =  < 0.001).

When combining the ADI-R and ADOS-2/BOSA at 36 months to give a research classification of autism, 14% of the NF1 cohort met criteria for autism compared to 0% of TD participants; this was also statistically not significant (X^2^ (1) = 2.71, *p* = 0.10) (Table 3).

## Discussion

To our knowledge, this is the first study to systematically investigate the developmental trajectories of cognitive and behavioural development in children with NF1 from 5 to 36 months of age using both objective and parental report measures. Our study suggests significantly lower trajectories of cognitive, motor, language and adaptive behaviour development in the NF1 group, with an overall mean difference that strengthens over developmental time and is clear by 24 months of age.

On the MSEL, lower scores are first evident at 5 months, but these differences are strengthened by 24 months and remain at 36 months. These results are consistent with a previous longitudinal study which showed significantly lower cognitive function in NF1 at 21 months of age, which remained at the follow-up evaluation at 40 months [17] . It is interesting to note that only subtle group differences in cognitive functioning were observed at 10 and 14 months, whilst behavioural differences were evident at 10 and 14 months. Our previous reports on the same cohort suggest differences arising in infancy in early auditory habituation and visual attention. Using an auditory habituation paradigm, Begum-Ali et al. found developmental differences in auditory processing in infants with NF1 at 10 and 14 months, possibly suggestive of alterations in early sensory processing and specialisation [19]. Such early-stage processing differences that typically present in the first year of life may represent the beginning of a series of compensatory and adaptive brain processes that trigger an alternative trajectory of subsequent development [35, 36] .

There was no significant difference between children with NF1 and TD children on the Socialization domain of the parent-rated VABS. It is possible that early social communication differences were less likely to be recognised by parents. The majority of the participants in this study had an inherited form of NF1. The parents themselves are at higher risk of having social communication and interaction difficulties, due to established higher rates of autism in individuals with NF1 [37] . However, this could also have been due to limited power related to the small sample size.

Based on the results of the parent rated VABS, there may be differences in the pace of developmental change in the NF1 group with periods of ‘catch-up’ over time. This is consistent with previous literature, which suggests that children with NF1 may fluctuate in their development, having periods where they improve in relation to typically developing peers, before experiencing delay again. For example, in a study by Wessel et al., 43 of the subjects had multiple cognitive assessments over time, and they moved between delayed and non-delayed groups [18] .

Our results indicate that levels of ADHD traits are higher in the NF1 group but were in the clinically significant range for only 10.5% at 36 months. Behavioural features of ADHD generally tend to peak in the school-age period, which has also been noted in NF1 with rates of ADHD as high as 50% [7]. Animal model studies suggest that dopaminergic dysfunction in NF1 contributes to attentional deficits [38]. Clinically, the ADHD symptomatology seen in NF1 is very similar to idiopathic ADHD. However, in-depth analyses of neurophysiological processes underlying attention difficulties in NF1 suggests differences in cognitive control processes [39]. Further understanding of these processes will be needed to move away from generic pharmacological intervention for ADHD and develop more personalised approaches. Buitelaar et al. review the emerging field of precision medicine in ADHD, identifying that there is a need to design treatments based on an individual patient’s genetic, biological or clinical features. Future areas of research include determining imaging or biological biomarkers that may predict clinical course and treatment response [40] .

Of note, only 3 participants at 36 months gained a T-score > 65, however all of these participants also met research criteria for autism, in keeping with previous research indicating that autism and ADHD often co-occur in children with NF1 [7]. There is also a possibility that measurement error could play a role, given the overlap in symptomatology between autism and ADHD. For example, Kochhar et al. recognise that children with ADHD have difficulties with social functioning, and therefore ADOS-2 scores could risk a false-positive autism diagnosis in this cohort [41]. However, Salley et al. concluded in their study of 209 children aged 3–18, with either autism, ADHD, both or no diagnosis, that the ADOS-2 can provide diagnostic symptom delineation between autism and ADHD for social communication and interaction deficits [42]. concluded in their study of 209 children

We hypothesised that higher proportions of the NF1 cohort would demonstrate autism traits on administered instruments at 24 and 36 months compared to TD participants. Almost half of the NF1 sample met instrumental threshold for autism at 24 months on the observer rated measures, but this was somewhat attenuated at 36 months (31%). When combining both the parental interview (ADI-R) and the observer rated measures (ADOS-2 or BOSA-MV), 14% of the NF1 sample met research classification for autism at 36 months. These results were statistically non-significant (most likely due to sample size), however they are consistent with previous studies which suggested rates of co-occurring autism in NF1 between 10 and 25% [6] .

Given that the autism behavioural phenotype in NF1 is broadly similar to idiopathic autism [6], it will be important to identify similarities and differences in early-stage markers in the two cohorts. Our research suggests that by 24 months of age, the NF1 cohort showed higher levels of autism traits compared with TD children. Our previous work has demonstrated that at 5, 10 and 14 months, developmental trajectories of social communication skills were similar between NF1 and TD groups [15]. In contrast, evidence suggests that social communication differences are detectable between 6 and 18 months of age in children who later go on to develop idiopathic autism [43]. Such differences may include non-orientation to their own name being called [44], reduced use of gesture [45] and reduced vocalisations [46]. Further research is required to understand why such behavioural signs of autism may emerge later for children with NF1 compared to children with idiopathic autism.

Consistent with a previous longitudinal study of NF1 children aged 21–40 months [17] we largely found no sex differences in the trajectories of cognitive, behavioural or social development. These sex differences may emerge at a later timepoint, as there are significant sex differences observed in cross-sectional studies in school-age children with NF1 [5, 47]. Males are more likely to demonstrate differences in learning and social functioning, mirroring the pattern seen in the general population [5, 47]. Longitudinal studies of infants with high familial likelihood of neurodevelopmental conditions such as autism or ADHD have found that females in general perform better than males in all dimensions of cognitive functioning [48]. Alternatively, sex differences may not have been noted in our sample due to the relatively small sample size.

In clinical practice, the proportion of children with inherited NF1 is approximately 50%. However, in our study, at least 91% of participants had an inherited pathogenic variant, necessitated by the early age at which assessments began. Many signs of NF1 present in later childhood [49], meaning that children with a sporadic pathogenic variant may be diagnosed later than children with a family history. It is hypothesised that having a parent with NF1 (who are themselves at higher likelihood of having autism, ADHD and cognitive difficulties) is likely to impact the cognitive, behavioural and social development of the child. Some studies have sought to clarify whether the mode of inheritance might explain some of the phenotypic variability in NF1. Biotteau et al. investigated whether the mode of transmission of the NF1 genetic variant (sporadic versus inherited) might explain cognitive differences in school-aged children [50]. IQ expression differed between groups, with children who had inherited NF1 performing less well. However, environmental factors also modulated cognitive ability, such as socioeconomic status [50]. Similarly, Hou et al. determined in their study of 88 children with NF1 that those who had parents with NF1 were more likely to have lower scores in performance IQ, writing, reading, working memory and attention than those whose parents did not have NF1 [51].

The clear emergence of differences between children with NF1 and TD at 24 months of age has clinical importance. It suggests that children with NF1 would benefit from screening and monitoring of their developmental progress from the age of 2, with proactive referral for early interventions considered by their clinical team. This would allow for the child’s development to be optimised prior to starting formal education. Research suggests that early intervention can modulate later behavioural manifestations of autism in children with idiopathic autism; Green et al. demonstrated that a parent-mediated social communication intervention from 7 months of age in children at high familial likelihood for autism showed a reduction in autism symptoms and improvement in social communication at 3 years of age [16]. Moreover, there is evidence to suggest that children with NF1 may gain progress with sufficient resources and time in an educational setting, highlighting the importance of early intervention to optimise educational outcomes [5] .

## Limitations

Methodological strengths of our study included tracking of a prospectively ascertained sample of children with NF1. Limitations include a relatively small sample size due to variable retention and rolling recruitment but the use of linear mixed modelling allowed for imputation of the missing data. The NF1 group was composed primarily of children with an inherited pathogenic variant, due to the early age at which assessments began. Our TD group showed a higher than expected proportion of participants reaching threshold for autism at 36 months on the ADOS-2/BOSA, however when combined with the ADI-R, none of the TD group was given a research classification of autism.

Due to limitations imposed by the COVID-19 pandemic, we utilised the BOSA for some participants, which was administered online and may not gather as accurate a picture of traits as ADOS-2. The BOSA-MV was utilised for 41% of participants with NF1 at 36 months compared to 11% at 24 months. This may explain the reduction in the percentage of children with NF1 that met autism criteria at 36 months.

Research thresholds using the ADI-R and ADOS/BOSA rather than gold-standard clinical best estimates were used for the autism classification. Gold standard clinical best estimates were not used because it would be best practice to combine parental report and direct observation with information from another setting, such as education. However it was beyond the scope of this study to collect educational information from the pre-school setting, therefore we utilised research instrumental thresholds.

The ADI-R subscale for Restrictive, Repetitive and Stereotyped behavior was not utilised in our research classifications of autism, which was based on criteria suggested by Risi et al. [34] Children with NF1 have previously been shown to have fewer restrictive, repetitive and stereotyped behaviours, compared to children with idiopathic autism and children diagnosed with other RASopathies such as Noonan syndrome and cardiofaciocutaneous syndrome [37, 52]. The mean score for the ADI-R subscale C was higher in children with NF1 compared to TD children, however it is possible that if gold-standard clinical judgement had been used, some of the children given a classification of autism may not in fact meet criteria specified by the DSM-5 which stipulates that two out of the four restrictive, repetitive behaviours and interests criteria must be met [53] .

## Conclusions

Our results collectively suggest that the NF1 brain development is atypical, with early-stage sensory processing difficulties seen in infancy with consolidated behavioural phenotypic differences by 24 months [15, 19]. Clinically, this highlights that developmental monitoring and referral for early interventions should be considered by the age of 2 in children with NF1. Intervention targeting neurocognitive modifiers such as executive attention or social engagement may ameliorate the impact of genetic or environmental vulnerabilities on the developing brain [47]. Future work should include replication of our findings in larger cohorts, investigating the similarities and differences in the developmental profiles seen in NF1 to other cohorts of infants at higher likelihood of common neurodevelopmental conditions such as autism and ADHD. This will inform potential intervention development.

## Supplementary Information

Additional file 1.

## Data Availability

Deidentified participant data is available through the STAARS network via data sharing procedures that comply with ethical requirements, due to the sensitive nature of the data collected. Available at: https://www.staars.org There is no known end date of data availability.

## Abbreviations

NF1: Neurofibromatosis type 1
ADHD: Attention deficit hyperactivity disorder
MSEL: Mullen scales of early learning
VABS: Vineland adaptive behavior scales
ADOS-2: Autism diagnostic observation schedule
BOSA-MV: Brief observation of symptoms of autism for minimally verbal children
ADI-R: Autism diagnostic interview-revised
CBCL: Child behavior checklist
