# Supplementary material for: Developmental trajectories in infants and pre-school children with Neurofibromatosis 1
Source: Mol Autism. 2024 Oct 15;15:45. doi: 10.1186/s13229-024-00621-5 (PMC11481376; doi:10.1186/s13229-024-00621-5)
Supplement: Supplementary file 1 — Additional file 1. [file 13229_2024_621_MOESM1_ESM.docx]

**Supplementary materials**

**Supplementary Figure 1** shows the number of NF1 and TD participants at each time point in the study. In the NF1 group 22% missed zero sessions, 34% missed one session, 26% missed two sessions, 9% missed three sessions and 9% missed four sessions. In the TD sample 52% missed zero sessions, 31% missed one session, 7% missed two sessions, 0% missed three sessions and 10% missed four sessions.

**Supplementary Figure 1:** Study numbers and attrition


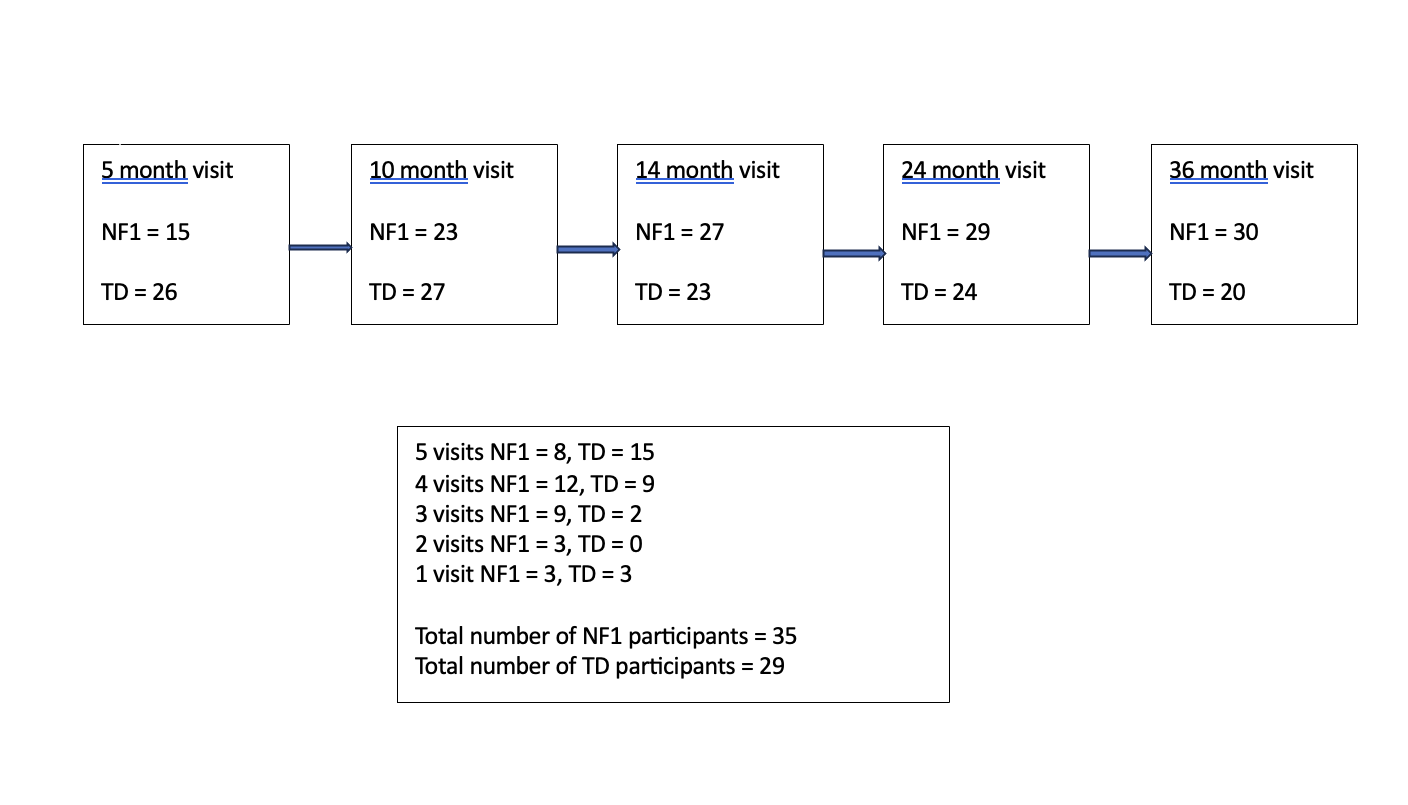


**Supplementary Table 1:** Descriptive statistics for MSEL and VABS

|  | **5 Months** | | | | **10 Months** | | | | **14 Months** | | | | **24 months** | | | | **36 months** | | | | | |
| --- | --- | --- | --- | --- | --- | --- | --- | --- | --- | --- | --- | --- | --- | --- | --- | --- | --- | --- | --- | --- | --- | --- |
|  | **TD group** | | **NF1 Group** | | **TD group** | | **NF1 Group** | | **TD group** | | **NF1 Group** | | **TD group** | | **NF1 Group** | | **TD group** | | **NF1 Group** | | | |
|  | ***n*** | ***Mean***  ***[SD]*** | ***n*** | ***Mean***  ***[SD]*** | ***n*** | ***Mean***  ***[SD]*** | ***n*** | ***Mean***  ***[SD]*** | ***n*** | ***Mean***  ***[SD]*** | ***n*** | ***Mean***  ***[SD]*** | ***n*** | ***Mean***  ***[SD]*** | ***n*** | ***Mean***  ***[SD]*** | ***n*** | ***Mean***  ***[SD]*** | ***n*** | ***Mean***  ***[SD]*** | | |
| **Mullen Scales of Early Learning T scores**  Gross Motor  Visual reception  Fine motor  Receptive language  Expressive language  Early Learning Composite Scores | 26  26  26  26  26  26 | 43.69 [9.41]  47.27 [6.33]  42.92 [10.46]  36.88 [11.28]  41.85 [7.45]  85.00[9.32] | 15  15  15  14  15  14 | 37.40 [9.72]  37.47 [11.53]  32.8 [6.99]  25.71 [9.31]  35.13 [6.35]  67.86 [9.46] | 27  27  27  27  27  27 | 34.89 [11.78]  48.85 [7.99]  51.63 [12.88]  39.26 [8.96]  36.85 [9.89]  88.89 [12.19] | 23  23  23  23  23  23 | 30.00 [10.25]  43.96 [8.08]  42.13 [11.29]  34.87 [7.73]  39.35 [12.56]  81.13 [10.70] | 23  23  23  23  23  23 | 36.74 [13.38]  35.09 [8.88]  49.65 [12.18]  32.87 [6.50]  37.09 [8.81]  78.78 [11.99] | 27  27  27  27  27  27 | 31.96 [12.75]  33.67 [5.17]  43.85 [11.07]  28.33 [6.01]  38.19 [10.18]  73.67 [7.30] | -  24  24  24  24  24 | -  59.63 [10.66]  56.00 [12.98]  57.67 [8.73]  55.42 [12.30]  114.25[17.91] | 26  27  27  27  27  27 | 35.04 [8.45]  33.11 [9.83]  33.89 [10.49]  33.85 [10.65]  34.52 [8.70]  81.48[17.91] | -  19  19  19  19  19 | -  67.58 [9.34]  64.68 [11.32]  65.84 [6.30]  60.84 [8.51]  129.05[11.75] | -  27  27  27  26  26 | -  40.63 [8.65]  31.22 [9.67]  35.89 [9.70]  39.50 [10.57]  75.88[13.45] | | |
| **Vineland Adaptive Behavior Scales Standard Scores**  Communication  Daily living skills  Socialisation  Motor  Adaptive Behavior composite | 22  22  23  21  19 | 94.95 [10.91]  86.32 [19.86]  98.70 [12.26]  88.67 [12.99]  90.63 [11.93] | 14  14  14  14  14 | 95.71 [14.04]  92.71 [18.26]  101.36 [14.21]  85.07 [13.37]  92.29 [11.13] | 21  22  22  21  20 | 92.86 [12.76]  102.45 [10.76]  98.77 [10.04]  95.76 [16.86]  96.80 [10.44] | 23  23  21  23  21 | 86.13 [14.91]  93.74 [9.14]  92.76 [14.59]  79.91 [14.86]  85.86 [12.85] | 19  18  18  18  17 | 96.11 [10.49]  93.44 [8.39]  94.22 [9.79]  101.67 [9.49]  95.29 [9.73] | 24  23  23  24  23 | 91.04 [10.08]  95.52 [10.85]  97.96 [9.43]  93.42 [11.14]  93.17 [9.36] | 22  22  22  22  22 | 112.14 [8.82]  108.36 [7.36]  104.64 [7.86]  102.41 [7.93]  108.05 [8.16] | 22  22  22  22  22 | 93.41 [7.5]  96.14 [9.25]  94.55 [7.71]  93.05 [8.67]  92.73 [5.78] | 12  12  12  12  12 | 105.08 [8.58]  104 [10.06]  103.25 [6.05]  92 [8.52]  100.92 [8.58] | 30  30  30  30  30 | 90.73 [14.23]  97.9 [14.67]  98.47 [15.78]  83.77 [13.13]  91.4 [14.62] | | |
| Missing data not included in n, mean or SD but kept in model for imputation | | | | | | | | | | | | |  |  |  |  |  | | |  |  |  |

**Supplementary Table 2:** Post-hoc T-test analysis for MSEL and VABS (Bonferroni corrected alpha value <0.01)

|  | **5 Months T-test**  **(NF1 group – TD group)** | **10 Months T-test**  **(NF1 group – TD group)** | **14 Months T-test**  **(NF1 group – TD group)** | **24 Months T-test**  **(NF1 group – TD group)** | **36 Months T-test**  **(NF1 group – TD group)** |
| --- | --- | --- | --- | --- | --- |
| **Mullen Scales of Early Learning T Scores**  Visual Reception  Fine Motor  Receptive Language  Expressive Language  Early Learning Composite Scores | t(39)=-3.53, p<0.001*  t(39)=-3.34, p=0.002*  t(38)=-3.17, p=0.003*  t(39)=-2.93, p=0.006*  t(38)=-5.52, p<0.001* | t(48)=-2.15, p=0.037  t(48)=-2.75, p=0.008*  t(48)=-1.84, p=0.072  t(48)= 0.79, p=0.436  t(48)=-2.37, p=0.022 | t(48)=-0.70, p=0.485  t(48)=-1.76, p=0.084  t(48)=-2.56, p=0.014  t(48)=0.40, p=0.688  t(48)=-1.85, p=0.07 | t (49)=-9.24, p<0.001*  t(49)=-6.72, p <0.001*  t(49)=-8.67, p <0.001*  t(49)=-7.06, p <0.001*  t(49)=-6.41, p <0.001* | t(44) =-10.07, p <0.001*  t(44)=-10.77, p <0.001*  t(44)=-11.80, p <0.001*  t(44)=-7.24, p <0.001*  t(44)=-13.80, p <0.001* |
| **Vineland Adaptive Behavior Scales Standard Scores**  Communication  Daily Living Skills  Socialization  Motor Skills  Adaptive Behaviour Composite | t(34)=0.182, p=0.857  t(34)=0.971, p=0.338  t(35)=0.603, p=0.550  t(33)=-0.793, p=0.433  t(31)=0.405, p=0.688 | t(42)=-1.601, p=0.117  t(43)=-2.933, p=0.005*  t(41)=-1.580, p=0.122  t(42)=0.585, p<0.001*  t(39)=-2.983, p<0.005* | t(41)=0.79, p=0.116  t(39)=0.67, p=0.507  t(39)=1.237, p=0.223  t(40)=-2.527, p=0.16  t(38)=-0.696, p=0.490 | t(42)=-7.59, p <0.001*  t(42)=-4.85, p <0.001*  t(42)=-4.30, p <0.001*  t(42)=-3.74, p <0.001*  t(42)=-7.19, p <0.001* | t(40)=-3.25, p <0.001*  t(40)=-1.32, p 0.195  t(40)=-1.02, p 0.316  t(40)=-2.00, p 0.052  t(40)=-2.11, p 0.042 |

**Supplementary Table 3:** Estimated marginal means (adjusted for maternal education as co-variate) for MSEL and VABS

|  | **5 Months** | | | | **10 Months** | | | | **14 Months** | | | | **24 months** | | | | **36 months** | | | | | |
| --- | --- | --- | --- | --- | --- | --- | --- | --- | --- | --- | --- | --- | --- | --- | --- | --- | --- | --- | --- | --- | --- | --- |
|  | **TD group** | | **NF1 Group** | | **TD group** | | **NF1 Group** | | **TD group** | | **NF1 Group** | | **TD group** | | **NF1 Group** | | **TD group** | | **NF1 Group** | | | |
|  | ***n*** | ***Estimated Marginal Means***  ***[Standard error]*** | ***n*** | ***Estimated Marginal Means***  ***[Standard error]*** | ***n*** | ***Estimated Marginal Means***  ***[Standard error]*** | ***n*** | ***Estimated Marginal Means***  ***[Standard error]*** | ***n*** | ***Estimated Marginal Means***  ***[Standard error]*** | ***n*** | ***Estimated Marginal Means***  ***[Standard error]*** | ***n*** | ***Estimated Marginal Means***  ***[Standard error]*** | ***n*** | ***Estimated Marginal Means***  ***[Standard error]*** | ***n*** | ***Estimated Marginal Means***  ***[Standard error]*** | ***n*** | ***Estimated Marginal Means***  ***[Standard error]*** | | |
| **Mullen Scales of Early Learning T scores**  Visual reception  Fine motor  Receptive language  Expressive language  Early Learning Composite Scores | 26  26  26  26  26 | 48.25 [1.75]  43.75 [2.30]  36.75 [1.77]  41.66 [1.98]  85.98 [2.65] | 15  15  14  15  14 | 38.02 [2.36]  29.66 [3.05]  25.27 [2.52]  32.77 [2.69]  64.53 [3.53] | 27  27  27  27  27 | 48.16 [1.66]  50.78 [2.19]  38.72 [1.67]  37.41 [1.87]  88.13 [2.53] | 23  23  23  23  23 | 43.71 [1.74]  41.28 [2.76]  34.73 [1.76]  39.39 [1.97]  80.15 [2.61] | 23  23  23  23  23 | 35.24 [1.75]  49.95 [2.30]  32.99 [1.77]  37.44 [1.98]  79.46 [2.65] | 27  27  27  27  27 | 34.41 [1.67]  43.56 [2.20]  27.70 [1.69]  37.80 [1.90]  73.36 [2.53] | 24  24  24  24  24 | 59.77 [1.75]  56.52 [2.30]  57.59 [1.77]  56.30 [1.98]  115.18 [2.65] | 27  27  27  27  27 | 33.61 [1.67]  34.82 [2.20]  33.70 [1.69]  34.76 [1.90]  82.20 [2.53] | 19  19  19  19  19 | 67.74 [1.96]  63.80 [2.55]  65.64 [1.99]  61.49 [2.22]  128.85 [2.91] | 27  27  27  26  26 | 40.34 [1.67]  31.32 [2.20]  35.86 [1.69]  39.89 [1.93]  75.46 [2.57] | | |
| **Vineland Adaptive Behavior Scales Standard Scores**  Communication  Daily living skills  Socialisation  Motor  Adaptive Behavior composite | 22  22  23  21  19 | 94.99 [2.52]  85.48 [2.65]  99.40 [2.31]  87.69 [2.56]  90.85 [2.32] | 14  14  14  14  14 | 96.99 [3.13]  89.35 [3.37]  103.57 [2.86]  85.15 [3.17]  92.42 [2.68] | 21  22  22  21  20 | 91.88 [2.52]  102.77 [2.60]  98.91 [2.31]  94.30 [2.57]  96.27 [2.28] | 23  23  21  23  21 | 87.38 [2.34]  93.98 [2.47]  92.59 [2.27]  76.88 [2.39]  86.01 [2.15] | 19  18  18  18  17 | 96.10 [2.57]  93.53 [2.79]  94.60 [2.45]  101.54 [2.69]  95.18 [2.37] | 24  23  23  24  23 | 90.15 [2.39]  94.99 [2.58]  97.53 [2.28]  91.15 [2.44]  92.21 [2.15] | 22  22  22  22  22 | 112.79 [2.46]  108.67 [2.58]  105.06 [2.30]  103.09 [2.51]  108.85 [2.19] | 22  22  22  22  22 | 92.82 [2.55]  95.59 [2.70]  94.02 [2.37]  90.37 [2.59]  91.17 [2.24] | 12  12  12  12  12 | 106.04 [3.14]  105.38 [3.38]  104.09 [2.88]  94.18 [3.19]  103.06 [2.71] | 30  30  30  30  30 | 91.82 [2.22]  96.57 [2.33]  98.09 [2.09]  81.20 [2.27]  90.37[1.99] | | |
|  | | | | | | | | | | | | |  |  |  |  |  | | |  |  |  |
